# Association of triglyceride glucose index with all-cause and cardiovascular mortality in the general population

**DOI:** 10.1186/s12933-023-02054-5

**Published:** 2023-11-22

**Authors:** Jiaqi Chen, Kangxiang Wu, Yiying Lin, Mingyuan Huang, Shanghe Xie

**Affiliations:** Department of Cardiology, The Second Affiliated Hospital and Yuying Children’s Hospital of Wenzhou Medical University, Yongzhong Street, Wenzhou, 325000 Zhejiang China

## Abstract

**Objective:**

The Triglyceride-glucose (TyG) index, a novel indicator of insulin resistance, has been associated with mortality from coronary artery diseases, ischemic stroke, and heart failure. In recent years, much emphasis has been placed on the relationship between the TyG index and mortality in the general population. However, the impact of age on the association between TyG and all-cause and cardiovascular mortality remains controversial. This study investigated the link between the TyG index and all-cause and cardiovascular mortality, emphasizing differences between older and non-older populations.

**Methods:**

Data from the National Health and Nutrition Examination Survey (2009–2018), encompassing 20,194 participants, were analyzed. The baseline TyG index was calculated as Ln [fasting triglycerides (mg/dL) × fasting glucose (mg/dL)/2]. Multivariate Cox proportional hazards regression models with restricted cubic splines and trend tests were employed to explore the association between the TyG index and all-cause and cardiovascular mortality, with emphasis on age-specific analysis. Subgroup analysis was conducted to examine whether the TyG index's association with mortality varied across different subgroups. Additionally, receiver operating characteristic curves were used to compare the predictive ability of the TyG index with the homeostasis model assessment of insulin resistance (HOMA-IR) for all-cause and cardiovascular mortality.

**Results:**

Over a median follow-up period of 105 months, all-cause mortality accounted for 13.345% of cases, and cardiovascular mortality accounted for 3.387%. Kaplan–Meier curves showed a significant increase in all-cause and cardiovascular mortality with higher TyG index values (both *P* for log-rank test < 0.001). However, during Cox proportional hazards regression analysis, no linear trend was observed between the TyG index and all-cause or cardiovascular mortality after adjusting for confounding factors (all-cause mortality: *P* for trend = 0.424; cardiovascular mortality: *P* for trend = 0.481). Restricted cubic splines revealed a non-linear association between the baseline TyG index and all-cause and cardiovascular mortality in the overall population (all-cause mortality: Non-linear *P* = 0.003; cardiovascular mortality: Non-linear *P* = 0.034). The effect of the TyG index was consistent across most subgroups in terms of all-cause and cardiovascular mortality, with no significant interaction with randomized factors (all-cause mortality: *P* for interaction = 0.077–0.940, cardiovascular mortality: *P* for interaction = 0.173–0.987), except for the age subgroup (all-cause mortality: *P* for interaction < 0.001, cardiovascular mortality:* P* for interaction < 0.001). Further age-specific analysis revealed that the association between the TyG index and all-cause and cardiovascular mortality remained significant in patients aged < 65 but not in those aged ≥ 65. Interestingly, a non-linear association was observed between the TyG index and all-cause mortality in individuals aged < 65 (Non-linear *P* = 0.011), while a linear relationship was observed with cardiovascular mortality, showing an upward trend (Non-linear* P* = 0.742, *P* for trend = 0.010). Further stratification according to age yielded similar results only in patients aged 45–64 (all-cause mortality: Non-linear *P* = 0.001 and cardiovascular mortality: Non-linear *P* = 0.902, *P* for trend = 0.015). Compared to HOMA-IR, the TyG index demonstrated superior predictive performance for all-cause and cardiovascular mortality (all-cause mortality: 0.620 vs. 0.524, *P* < 0.001; cardiovascular mortality: 0.623 vs. 0.537, *P* < 0.001).

**Conclusions:**

This study established a significant association between the TyG index and all-cause and cardiovascular mortality in the general population, particularly among individuals aged < 65. Notably, a non-linear association with all-cause mortality was observed in those aged < 65, while a linear relationship with cardiovascular mortality was found.

**Supplementary Information:**

The online version contains supplementary material available at 10.1186/s12933-023-02054-5.

## Background

Insulin resistance (IR) is characterized by reduced sensitivity to the effects of insulin [1]. While the euglycemic hyperinsulinemic clamp and intravenous glucose tolerance testing are considered the gold standard for assessing IR, their invasive and costly nature limits their clinical utility [2]. Currently, the homeostasis model assessment of insulin resistance (HOMA-IR) is widely used to assess IR, but this model has limitations for patients receiving insulin treatment or those with non-functioning beta cells [2, 3]. The novel triglyceride glucose (TyG) index offers a practical solution to these challenges [3, 4]. Numerous studies have demonstrated that the TyG index outperforms HOMA-IR in evaluating IR in both diabetic and non-diabetic patients [4].

It is now understood that patients with insulin resistance are at increased risk of various metabolic disorders, including abnormalities in blood sugar, blood lipids, and blood pressure [5]. Consequently, numerous investigations are currently underway to explore the relationship between the TyG index and conditions such as cardiovascular and cerebrovascular diseases and their prognoses [6–9]. However, studies involving the general population have produced inconsistent findings regarding the associations between the TyG index and all-cause and cardiovascular mortality. Some studies have reported no significant relationship between the TyG index and all-cause or cardiovascular mortality [10], while others documented a positive correlation [11] or a U-shaped relationship [12]. Accordingly, the controversy surrounding the TyG index has hindered its clinical applicability. Indeed, further exploration of the links between the TyG index and all-cause and cardiovascular mortality is crucial to promote its clinical use and enhance overall survival.

Current evidence suggests that the TyG index is influenced by various factors, including age and fasting status. Previous studies have revealed that younger patients tend to have higher TyG index levels [13, 14]. Younger individuals with elevated TyG levels may be more susceptible to IR-related diseases and mortality [15], as demonstrated in patients with cardiovascular disease and stroke [15, 16]. These findings indicate that individuals under 65 years old with higher TyG levels are at a greater risk of all-cause mortality related to cardiovascular disease and ischemic stroke. However, earlier research has also suggested that the association between the TyG index and mortality is more pronounced among older participants [17]. The impact of age on the relationship between the TyG index and all-cause and cardiovascular mortality remains a subject of controversy. We hypothesize that age plays a significant role in the predictive value of the TyG index for all-cause and cardiovascular mortality in the general population, thus building on and extending previous research. Therefore, this retrospective study aims to assess the predictive utility of the TyG index for all-cause and cardiovascular mortality in the general population and to investigate the potential influence of age on the relationship between TyG and all-cause and cardiovascular mortality.

## Methods

### Study population

**Fig. 1:**
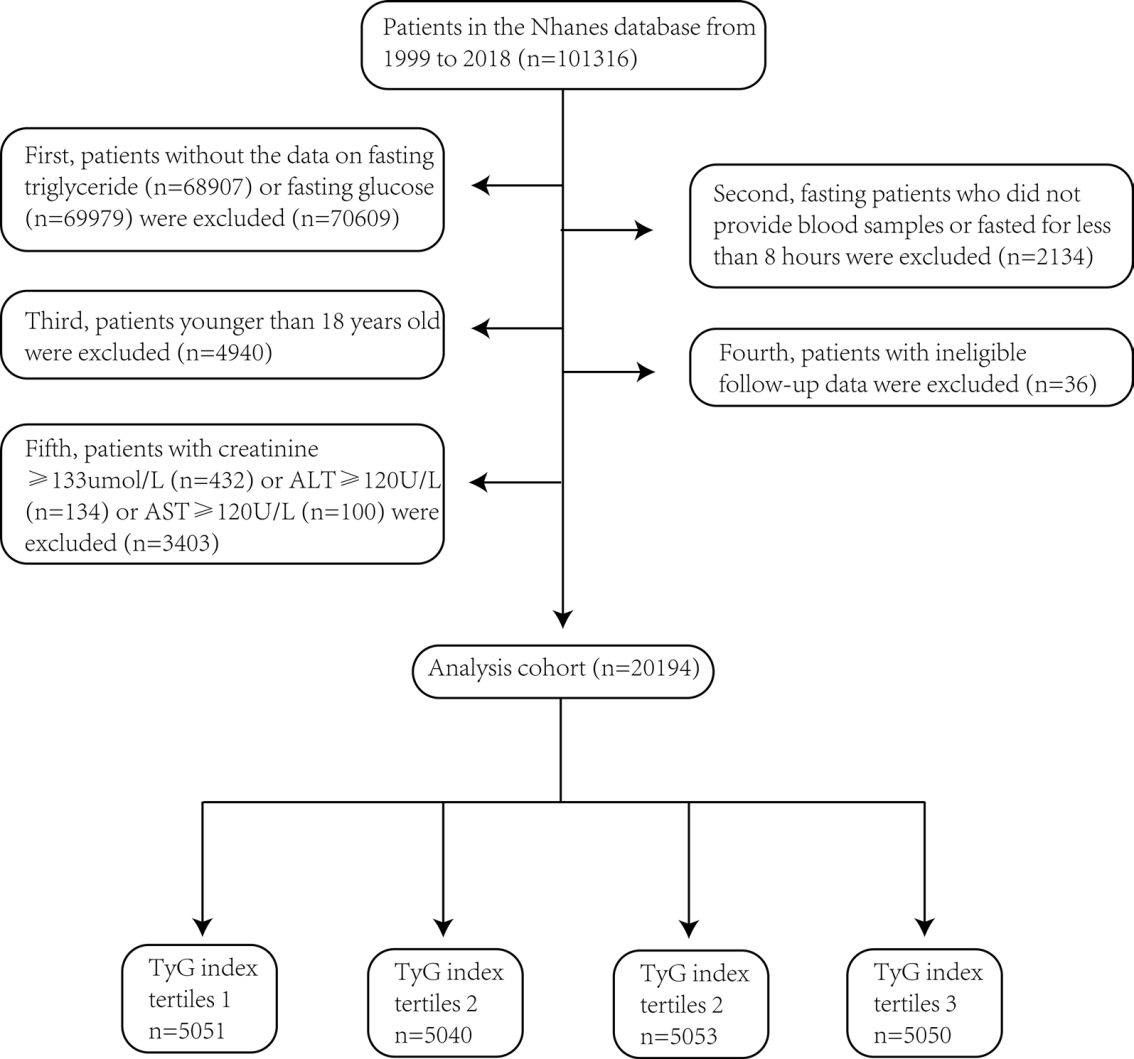
Process for inclusion of trial patients. *TyG Index* triglyceride glucose index, *ALT* alanine transaminase, *AST* aspartate transaminase

Data for this study were sourced from the National Health and Nutrition Examination Survey (NHANES), a nationally representative survey of nutrition and health conditions in the United States (https://wwwn.cdc.gov/nchs/nhanes/Default.aspx). NHANES was approved by the NCHS Research Ethics Review Board. Our investigation into the association between the TyG index and all-cause and heart disease-specific mortality employed NHANES data spanning from 1999 to 2018. The following groups of patients were excluded from the study: (1) Patients without fasting triglyceride and fasting glucose data. (2) Fasting patients who did not provide blood samples or fasted for less than 8 h. (3) Patients under the age of 18. (4) Patients with ineligible follow-up data. (5) Patients with renal insufficiency (blood creatinine ≥ 133 μmol/L) or severe liver disease (alanine or aspartate aminotransferase ≥ 120U/L). The final study population included 20,194 participants. The patient selection process is illustrated in Fig. 1.

### Data collection

Our study incorporated four main categories of covariates: (1) demographic characteristics, which encompassed age, gender, race, smoking status (current smoker: currently smoking and having smoked at least 100 cigarettes; former smoker: having smoked at least 100 cigarettes but not currently smoking; never smoker: having smoked fewer than 100 cigarettes), and education; (2) vital signs, including BMI, systolic and diastolic blood pressure; (3) laboratory parameters, such as alanine aminotransferase, aspartate aminotransferase, creatinine, triglycerides, cholesterol, uric acid, fasting blood glucose, insulin, glycohemoglobin, white blood cell count, platelet count, and red blood cell count; (4) comorbidities, including heart failure, diabetes, stroke, coronary artery disease, and hypertension; (5) medication usage, encompassing lipid-lowering agents (statins and fibrates) and hypoglycemic agents. The TyG index was calculated as Ln [fasting triglycerides (mg/dL) × Fasting glucose (mg/dL)/2] [4]. Blood samples were collected after fasting for a minimum of 8 h. The homeostatic model assessment insulin resistance index (HOMA-IR) was calculated as fasting insulin (μU/mL) × fasting plasma glucose (mg/dL)/405 [18, 19].

### Clinical outcomes

The primary endpoint of this study was all-cause mortality, with the secondary endpoint being cardiovascular mortality. Follow-up commenced on the date of the interview and concluded on the date of death or the conclusion of the mortality period (December 31, 2019).

### Statistical analysis

Study population characteristics were stratified based on TyG index quartiles, with categorical variables presented as survey-weighted percentages and continuous variables as survey-weighted means. The four groups were compared using the variance analysis or Kruskal–Wallis test for continuous variables and the χ^2^ test for categorical variables. Statistical significance was determined by a two-tailed P value of 0.05.

The specific association between the TyG index and all-cause and cardiovascular mortality was examined using multivariate Cox proportional hazards models. Three sets of models were constructed. The Crude model included only the TyG index. The Adjust I model included demographic characteristics (age, sex, and race). The Adjust II model incorporated additional adjustments for education, smoking, body measurements (BMI, SBP, DBP), laboratory parameters (cholesterol, uric acid), comorbidities (diabetes mellitus), and medication use (lipid-lowering agents and hypoglycemic agents). To explore the relationship between the TyG index and mortality, Cox proportional hazards regression models with restricted cubic splines and trend tests were conducted.

Stratified analysis was carried out for significant covariates, considering potential effect modifiers such as age, gender, body mass index, smoking, education, HbA1c (glycohemoglobin), uric acid, hypertension, heart failure, stroke, and coronary artery disease.

Receiver operating characteristic curves (ROC) were generated to compare the predictive abilities of the TyG index and HOMA-IR for all-cause and cardiovascular mortality.

## Result

### Characteristics of the population stratified by triglyceride glucose index

As presented in Table 1, baseline patient characteristics were stratified by TyG index quartiles (Q): Q1: < 8.148; Q2: 8.148–8.569; Q3: 8.570–9.019; Q4: ≥ 9.020. The mean TyG index levels in these quartiles were 7.811 ± 0.271, 8.365 ± 0.122, 8.783 ± 0.130, and 9.499 ± 0.471, respectively. In comparison with patients with lower TyG index values, those in the higher TyG index group tended to be male, older, smokers, have lower educational levels, use lipid-lowering agents and hypoglycemic agents, and exhibit a higher prevalence of comorbidities, including heart failure, coronary artery disease, diabetes, hypertension, and stroke. Moreover, the higher TyG index group was associated with higher BMI, SBP, DBP, HOMA-IR, and blood indicators, including ALT, AST, creatinine, TC, TG, uric acid, fasting glucose, glycosylated hemoglobin, insulin, white blood cell count, red blood cell count, and platelet count. With increasing TyG index, both all-cause mortality (6.652% vs. 12.222% vs. 15.417% vs. 19.089%, *P* < 0.001) and heart disease-specific mortality (1.425% vs. 3.095% vs. 3.859% vs. 5.168%, *P* < 0.001) exhibited a gradual increase.

**Table 1:** Characteristics of participants in the NHANES (2009–2018) by depression status

Variables	TyG index levels				*P*-value
	Q1	Q2	Q3	Q4	
Participants, No	5051	5040	5053	5050	
TyG index	7.811 ± 0.271	8.365 ± 0.122	8.783 ± 0.130	9.499 ± 0.471	< 0.001
Clinical parameters					
Age, years	39 ± 18	47 ± 19	50 ± 19	53 ± 17	< 0.001
Gender, n (%)					< 0.001
Male	2047 (40.527)	2376 (47.143)	2521 (49.891)	2726 (53.980)	
Female	3004 (59.473)	2664 (52.857)	2532 (50.109)	2324 (46.020)	
Race, n (%)					< 0.001
Mexican American	684 (13.542)	869 (17.242)	1025 (20.285)	1218 (24.119)	
Other Hispanic	330 (6.533)	385 (7.639)	470 (9.301)	429 (8.495)	
Non-Hispanic White	1812 (35.874)	2207 (43.790)	2315 (45.814)	2428 (48.079)	
Non-Hispanic Black	1676 (33.182)	1110 (22.024)	734 (14.526)	533 (10.554)	
Other Race	549 (10.869)	469 (9.306)	509 (10.073)	442 (8.752)	
Smoking, n (%)					< 0.001
Never smoker	2924 (64.662)	2633 (56.105)	2586 (53.243)	2388 (48.272)	
Former smoker	838 (18.532)	1096 (23.354)	1273 (26.210)	1495 (30.220)	
Current smoker	760 (16.807)	964 (20.541)	998 (20.548)	1064 (21.508)	
Vital signs					
BMI, kg/m^2^	26.21 ± 6.38	27.96 ± 6.67	29.55 ± 6.77	30.96 ± 6.39	< 0.001
DBP, mmHg	67 ± 12	69 ± 13	71 ± 13	72 ± 14	< 0.001
SBP, mmHg	118 ± 17	123 ± 19	126 ± 19	129 ± 20	< 0.001
Education, n (%)					< 0.001
Less than high school	1129 (22.365)	1306 (25.933)	1456 (28.843)	1620 (32.117)	
High school or equivalent	1332 (26.387)	1318 (26.172)	1245 (24.663)	1236 (24.504)	
College or above	2587 (51.248)	2412 (47.895)	2347 (46.494)	2188 (43.378)	
Laboratory parameters					
ALT, U/L	20 ± 10	22 ± 12	25 ± 14	28 ± 16	< 0.001
AST, U/L	23 ± 9	23 ± 9	24 ± 9	25 ± 10	< 0.001
Creatinine, umol/L	72.3 ± 17.3	74.3 ± 18.4	74.5 ± 19.0	75.1 ± 19.7	< 0.001
TC, mg/dl	172 ± 34	189 ± 38	200 ± 39	213 ± 47	< 0.001
TG, mg/dl	55 ± 14	89 ± 14	129 ± 24	245 ± 171	< 0.001
Uric acid, mg/dl	4.9 ± 1.2	5.3 ± 1.3	5.6 ± 1.4	5.8 ± 1.5	< 0.001
Fasting Glucose, mg/dl	93 ± 10	99 ± 13	104 ± 19	129 ± 57	< 0.001
HbA1c, (%)	5.3 ± 0.5	5.4 ± 0.6	5.6 ± 0.7	6.3 ± 1.7	< 0.001
Insulin (uU/mL)	8.29 ± 6.95	10.51 ± 9.42	13.50 ± 13.37	18.06 ± 19.56	< 0.001
White blood cell count, 1000 cells/uL	6.2 ± 1.9	6.7 ± 2.8	7.1 ± 2.1	7.4 ± 2.2	< 0.001
Red blood cell count, 1000 cells/uL	4.64 ± 0.49	4.71 ± 0.51	4.76 ± 0.51	4.82 ± 0.52	< 0.001
Platelet count, 1000 cells/uL	246 ± 64	252 ± 67	257 ± 68	256 ± 67	< 0.001
HOMA-IR	1.942 ± 1.723	2.620 ± 2.930	3.580 ± 4.184	6.007 ± 8.946	< 0.001
Drug use					
Lipid-lowering agents					< 0.001
No	1631 (80.983)	1925 (74.296)	1979 (69.610)	2118 (64.085)	
Yes	383 (19.017)	666 (25.704)	864 (30.390)	1187 (35.915)	
Hypoglycemic agent					< 0.001
No	1891 (93.893)	2356 (90.930)	2419 (85.086)	2226 (67.352)	
Yes	123 (6.107)	235 (9.070)	424 (14.914)	1079 (32.648)	
Comorbidities					
HF, n (%)					< 0.001
No	4227 (97.893)	4489 (97.101)	4584 (95.679)	4639 (94.500)	
Yes	91 (2.107)	134 (2.899)	207 (4.321)	270 (5.500)	
DM, n (%)					< 0.001
No	4856 (96.235)	4701 (93.292)	4477 (88.636)	3709 (73.489)	
Borderline	55 (1.090)	77 (1.528)	103 (2.039)	139 (2.754)	
Yes	135 (2.675)	261 (5.180)	471 (9.325)	1199 (23.757)	
Stroke, n (%)					< 0.001
No	4222 (97.754)	4491 (96.935)	4632 (96.259)	4717 (95.660)	
Yes	97 (2.246)	142 (3.065)	180 (3.741)	214 (4.340)	
CAD, n (%)					< 0.001
No	91 (2.107)	134 (2.899)	207 (4.321)	270 (5.500)	
Yes	4227 (97.893)	4489 (97.101)	4584 (95.679)	4639 (94.500)	
HTN, n (%)					< 0.001
No	4050 (80.597)	3582 (71.540)	3226 (64.122)	2822 (56.092)	
Yes	975 (19.403)	1425 (28.460)	1805 (35.878)	2209 (43.908)	
Outcomes					
All-cause mortality, n (%)	336 (6.652)	616 (12.222)	779 (15.417)	964 (19.089)	< 0.001
Cardiovascular mortality, n (%)	72 (1.425)	156 (3.095)	195 (3.859)	261 (5.168)	< 0.001

*TyG index* triglyceride glucose index, *BMI* body mass index, *DBP* diastolic pressure, *SBP* systolic pressure, *ALT* alanine transaminase, *AST* aspartate transaminase, *TC* cholesterol, *TG* triglyceride, *HbA1c* glycohemoglobin, *HOMA-IR* Homeostasis model assessment of insulin resistance, *HF* heart failure, *DM* diabetes mellitus, *CAD* coronary artery disease, *HTN* hypertension

### Kaplan–Meier survival analysis curves for all-cause and cardiovascular mortality according to triglyceride glucose index

**Fig. 2:**
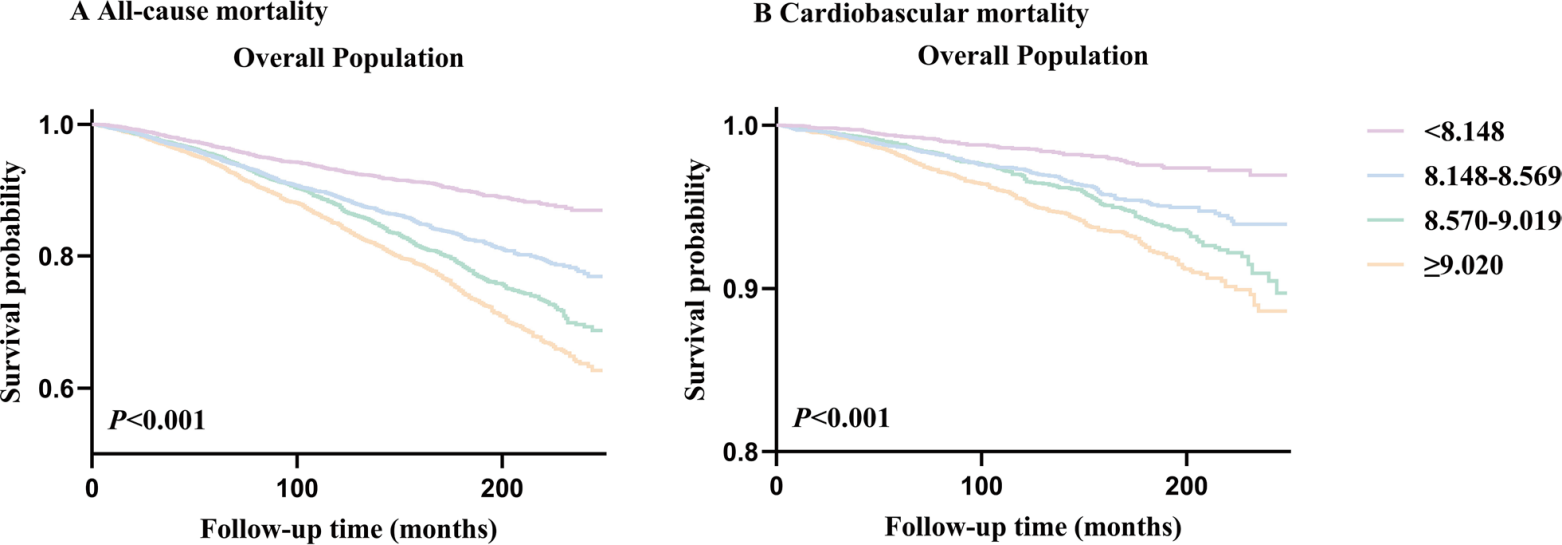
Kaplan–Meier survival analysis curves for all-cause and cardiovascular mortality. TyG index quartile (Q): Q1: < 8.148; Q2:8.148–8.569; Q3:8.570–9.019; Q4: ≥ 9.020. A Kaplan–Meier analysis for mortality among TyG index groups in **A** all-cause mortality, **B** cardiovascular mortality

During 2,441,212 person-months of follow-up (median follow-up of 105 months), there were 2,695 incident cases of all-cause mortality and 684 incident cases of cardiovascular mortality. The mortality across TyG index groups is illustrated in Fig. 2. A significant difference in mortality was observed among these groups (All-cause mortality:* P* for log-rank test < 0.001; cardiovascular mortality:* P* for log-rank test < 0.001) in the overall population (Fig. 2A, B).

### Associations of triglyceride glucose index with all-cause mortality and cardiovascular mortality

Cox proportional hazard analysis revealed a significant association between the TyG index and both all-cause and cardiovascular mortality in the crude model [All-cause mortality: HR (95% CI) 1.561 (1.485, 1.640), *P* < 0.001; cardiovascular mortality: HR (95% CI) 1.669 (1.516, 1.838), P < 0.001] and the adjusted models [Adjusted I: All-cause mortality: HR (95% CI) 1.168 (1.098, 1.244), *P* < 0.001; cardiovascular mortality: HR (95% CI) 1.319 (1.168, 1.490), P < 0.001; Adjusted II: all-cause mortality: HR (95% CI) 1.160 (1.062, 1.267), *P* < 0.001; cardiovascular mortality: HR (95% CI) 1.213 (1.020, 1.441), *P* = 0.029] when the TyG index was considered a continuous variable (Table 2).

**Table 2:** HR (95% CI) for outcomes across groups of TyG index

	Crude		Adjust I		Adjust II	
	HR (95% CI)	*P*-value	HR (95% CI)	*P*-value	HR (95% CI)	*P*-value
All-cause mortality						
TyG index						
Continuous	1.561 (1.485, 1.640)	< 0.001	1.168 (1.098, 1.244)	< 0.001	1.160 (1.062, 1.267)	< 0.001
Quartiles						
Q1	1		1		1	
Q2	1.706 (1.494, 1.949)	< 0.001	0.884 (0.773, 1.011)	0.072	0.852 (0.719, 1.011)	0.066
Q3	2.128 (1.872, 2.418)	< 0.001	0.908 (0.797, 1.035)	0.148	0.944 (0.798, 1.117)	0.504
Q4	2.628 (2.321, 2.975)	< 0.001	1.048 (0.922, 1.191)	0.472	0.974 (0.819, 1.158)	0.762
*P* for trend		< 0.001		0.046		0.424
Cardiovascular mortality						
TyG index						
Continuous	1.669 (1.516, 1.838)	< 0.001	1.319 (1.168, 1.490)	< 0.001	1.213 (1.020, 1.441)	0.029
Quartilies						
Q1	1		1		1	
Q2	2.017 (1.526, 2.667)	< 0.001	1.010 (0.762, 1.338	0.945	0.943 (0.660, 1.347)	0.747
Q3	2.489 (1.900, 3.262)	< 0.001	1.048 (0.796, 1.379)	0.738	0.983 (0.690, 1.401)	0.737
Q4	3.326 (2.562, 4.317)	< 0.001	1.330 (1.018, 1.737)	0.037	1.063 (0.743, 1.522)	0.481
*P* for trend		< 0.001		0.005		0.481

Adjust I: age, gender and race were adjusted

Adjust II: age, gender, race, BMI, SBP, DBP, TC, uric acid, DM, education, smoking, lipid-lowering agents and hypoglycemic agent were adjusted

Footnote TyG index quartile (Q): Q1: < 8.148; Q2:8.148–8.569; Q3:8.570–9.019; Q4: ≥ 9.020

*TyG Index* triglyceride glucose index, *BMI* body mass index, *DBP* diastolic pressure, *SBP* systolic pressure, *TC* cholesterol, *DM* diabetes mellitus, *HR* Hazard Ratio

In the crude and Adjust I models, there were upward trends between the TyG index and both all-cause and cardiovascular mortality (Table 2, both *P* for trend < 0.05). However, these results were not consistently observed in multivariate Cox proportional hazard analysis of the TyG index and all-cause and cardiovascular mortality in Model II (Table 2, all-cause mortality: *P* for trend = 0.424; cardiovascular mortality: *P* for trend = 0.481).

**Fig. 3:**
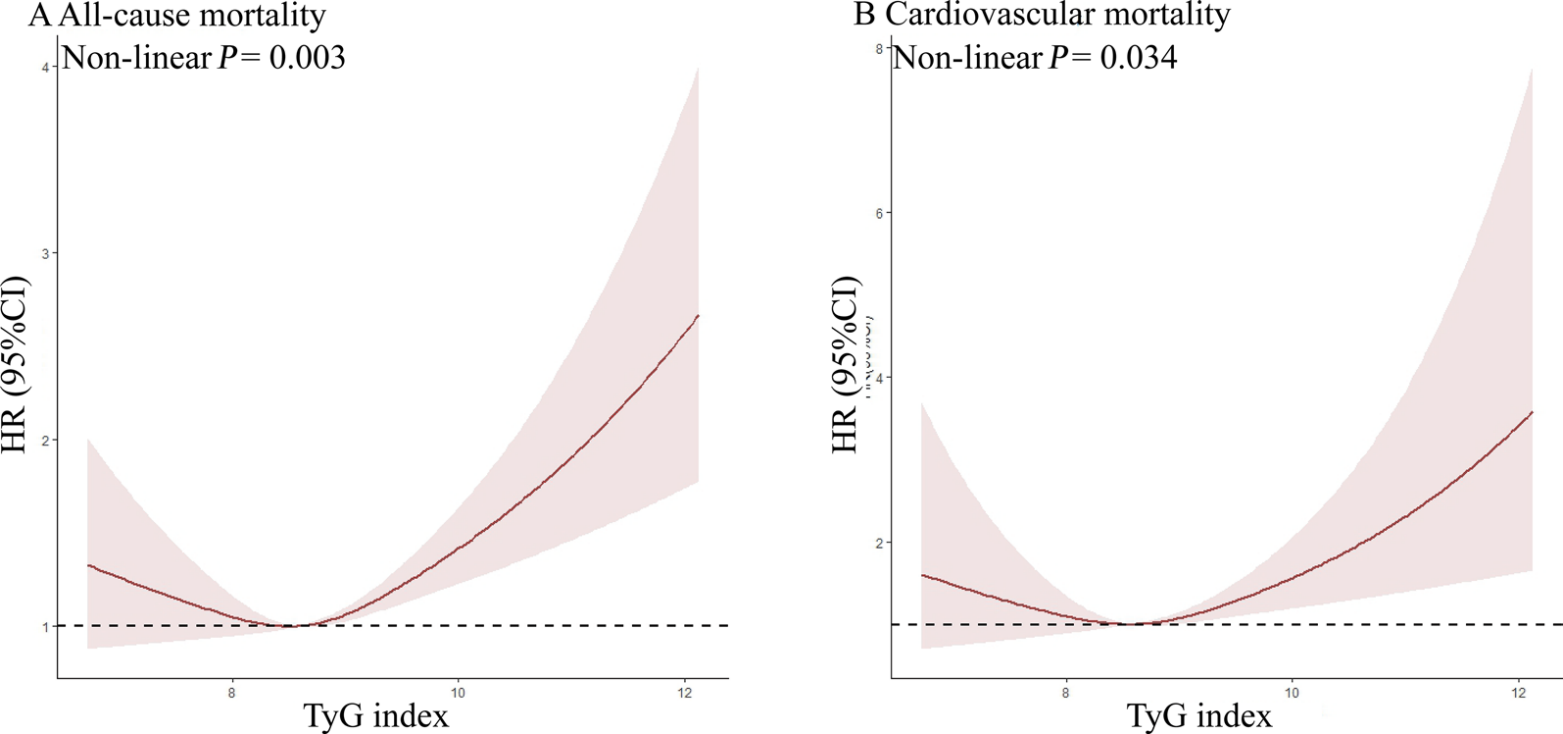
Association between TyG index and all-cause and cardiovascular mortality in the general population. **A** all-cause mortality, **B** cardiovascular mortality. Each hazard ratio was computed with a TyG index level of A 8.757 and B 8.975 as the reference. Adjusted for age, gender, race, BMI, SBP, DBP, TC, uric acid, DM, education, smoking, lipid-lowering agents and hypoglycemic agent. *TyG Index* triglyceride glucose index, *BMI* body mass index, *DBP* diastolic pressure, *SBP* systolic pressure, *TC* cholesterol, *DM* diabetes mellitus, *HR* Hazard Ratio

Given that multivariate Cox proportional hazard analysis suggested a non-linear relationship between the baseline TyG index and both all-cause and cardiovascular mortality, restricted cubic splines analysis was employed to further investigate this association. The adjusted restricted cubic spline plots displayed non-linear associations between TyG index and both all-cause (Fig. 3A, Non-linear* P* = 0.003) and cardiovascular mortality (Fig. 3B, Non-linear* P* = 0.034).

### Subgroup analysis of the association between TyG index and all-cause and cardiovascular mortality

**Fig. 4:**
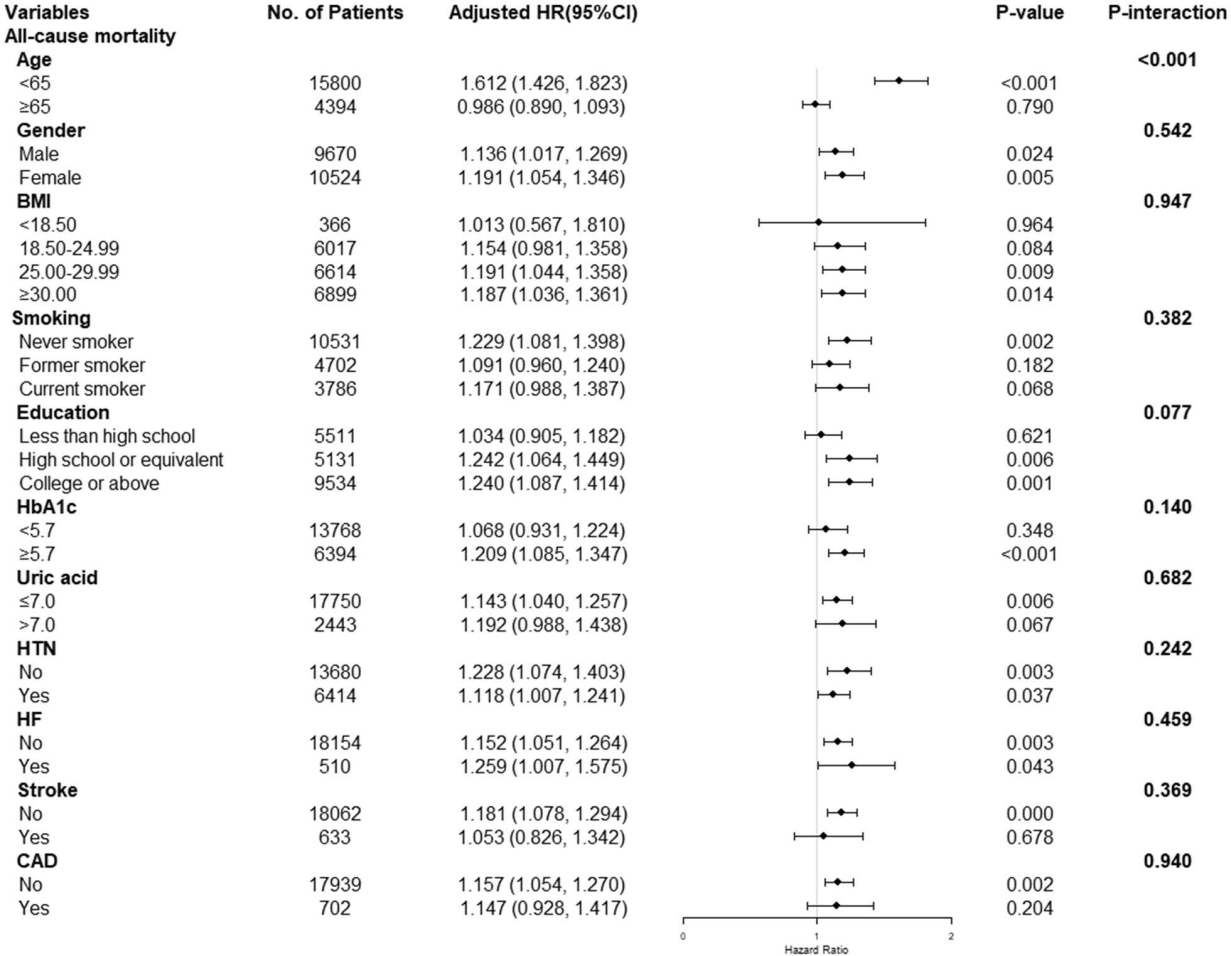
Subgroup analysis of the association between TyG index and all-cause mortality. Adjusted for age, gender, race, BMI, SBP, DBP, TC, uric acid, DM, education, smoking, lipid-lowering agents and hypoglycemic agent, except the subgroup factors themselves. *TyG Index* triglyceride glucose index, *BMI* body mass index, *DBP* diastolic pressure, *SBP* systolic pressure, *TC* cholesterol, *DM* diabetes mellitus, *HR* Hazard Ratio

**Fig. 5:**
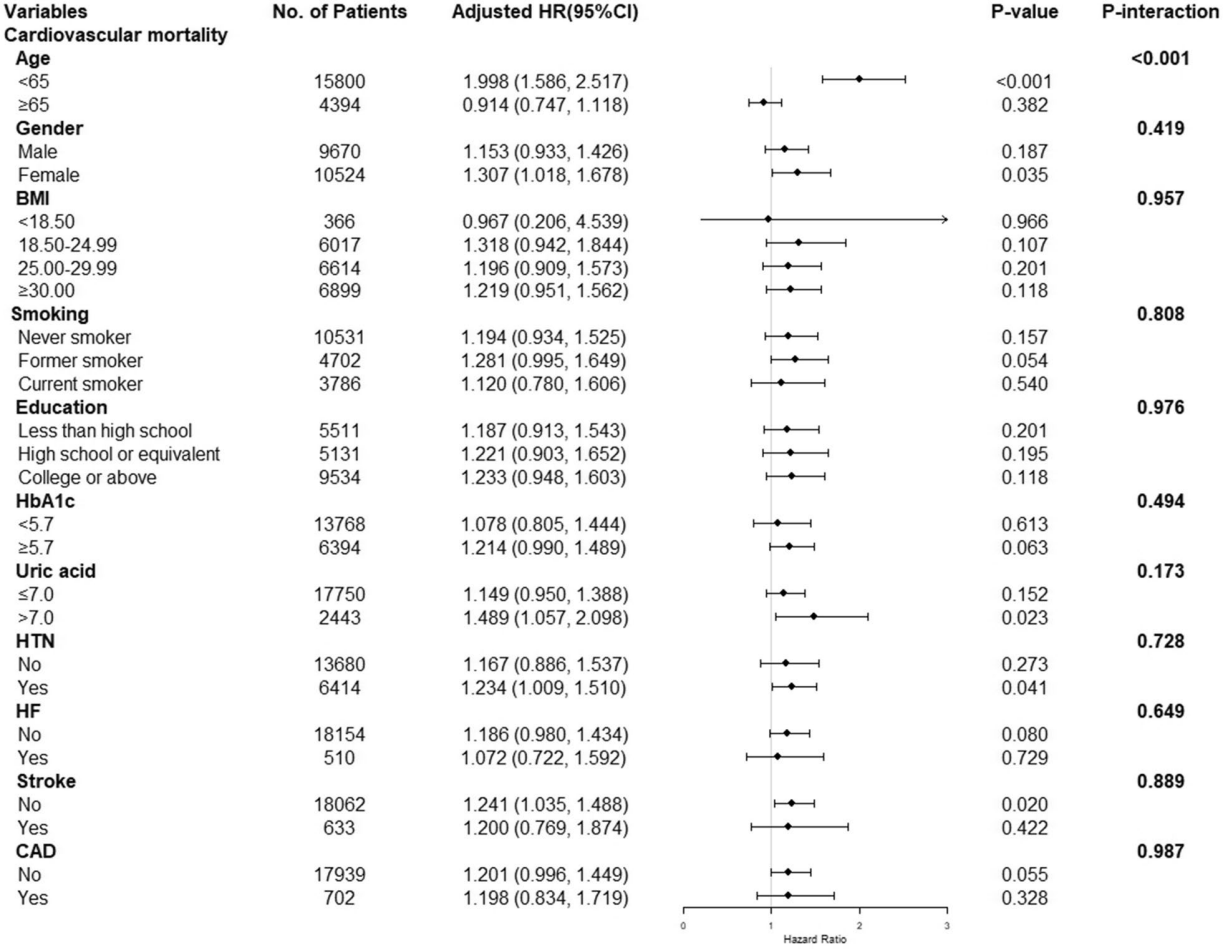
Subgroup analysis of the association between TyG index and cardiovascular mortality. Adjusted for age, gender, race, BMI, SBP, DBP, TC, uric acid, DM, education, smoking, lipid-lowering agents and hypoglycemic agent, except the subgroup factors themselves. *TyG Index* triglyceride glucose index, *BMI* body mass index, *DBP* diastolic pressure, *SBP* systolic pressure, *TC* cholesterol, *DM* diabetes mellitus, *HR* Hazard Ratio

To assess the impact of the TyG index on the primary endpoints, stratification was conducted according to age, gender, body mass index, smoking, education, HbA1c, uric acid, hypertension, heart failure, stroke, and coronary artery disease (Figs. 4, 5). Except for the age subgroup (age subgroup: all-cause mortality: *P* for interaction < 0.001, cardiovascular mortality: *P* for interaction < 0.001), there was no significant interaction in most subgroups (other subgroups: all-cause mortality: *P* for interaction = 0.077–0.940, cardiovascular mortality: *P* for interaction = 0.173–0.987). The TyG index was closely related to all-cause and cardiovascular mortality in patients aged < 65 (all-cause mortality: HR (95% CI) 1.612 (1.426, 1.823), *P* < 0.001; cardiovascular mortality: HR (95% CI) 1.998 (1.586, 2.517),* P* < 0.001), but not in patients aged ≥ 65 (All-cause mortality: HR (95% CI) 0.986 (0.890, 1.093), *P* = 0.790; cardiovascular mortality: HR (95% CI) 0.914 (0.747, 1.118),* P* = 0.382).

### Kaplan–Meier survival analysis curves for all-cause and cardiovascular mortality according to triglyceride glucose index and stratified by age

**Fig. 6:**
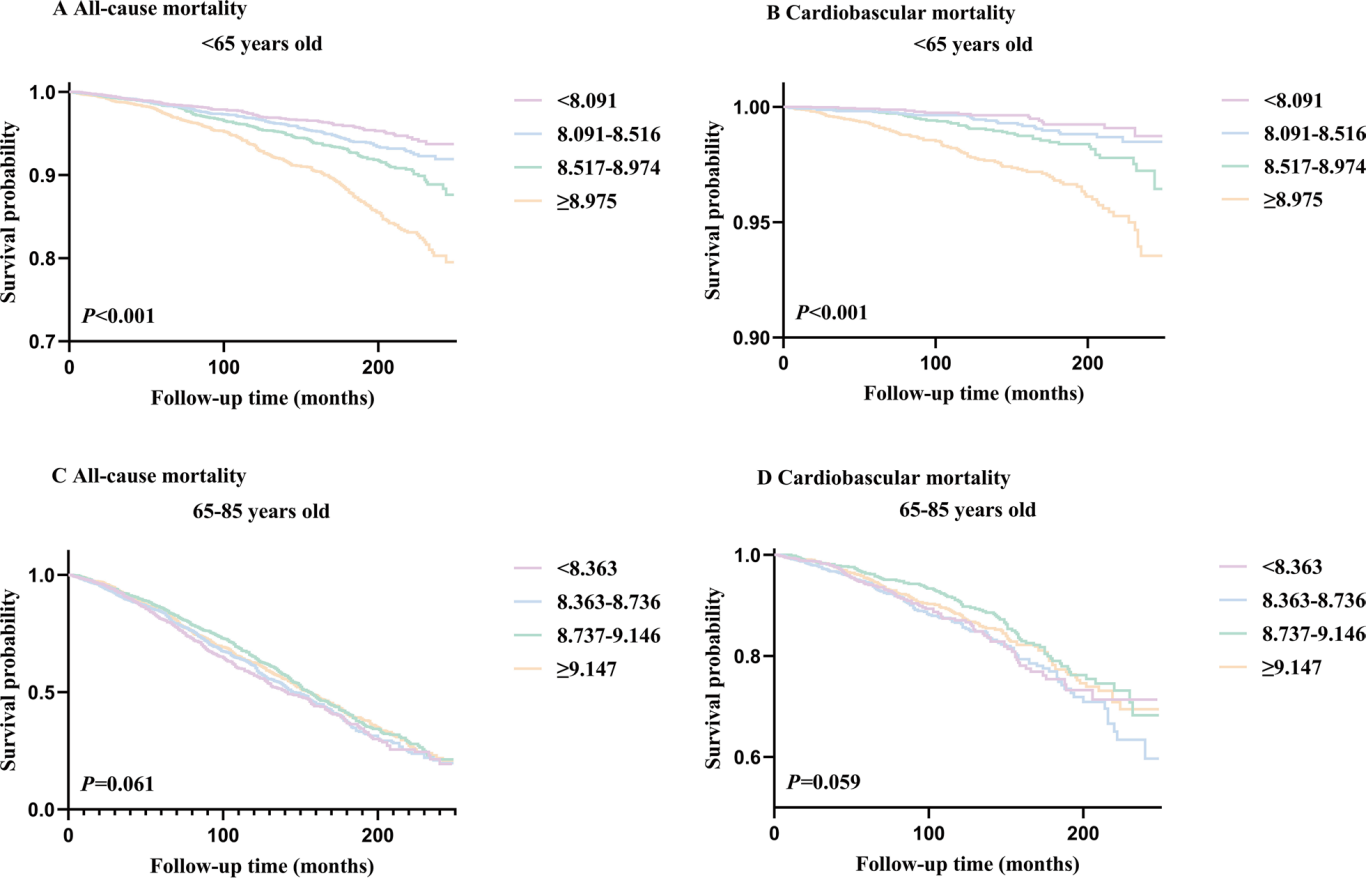
Kaplan–Meier survival analysis curves for all-cause and cardiovascular mortality after stratification by age. A Kaplan–Meier analysis for all-cause mortality among TyG index groups in **A**  < 65 years old, **C**  ≥ 65 years old. A Kaplan–Meier analysis for cardiovascular mortality among TyG index groups in **B**  < 65 years old, **D**  ≥ 65 years old

We grouped the study population based on age (< 65, ≥ 65) and divided these two groups into four quartiles to generate survival curves. Interestingly, age-stratified survival curves demonstrated that significant differences in mortality rates among groups persisted in the population aged < 65 (All-cause mortality: *P* for log-rank test < 0.001; cardiovascular mortality: *P* for log-rank test < 0.001, Fig. 6A, B). However, the differences in mortality among patients aged ≥ 65 did not reach statistical significance (All-cause mortality: *P* for log-rank test = 0.061; cardiovascular mortality: *P* for log-rank test = 0.059, Fig. 6C, D).

Further, the study population was grouped according to age (< 25, 25–44, 45–64, 65–85), and survival curves were plotted based on their respective quartiles. Significant differences in mortality among groups persisted in the population aged 25–44 and 45–64 (both *P* for log-rank test < 0.05) (Additional file 1: Figure S1A, B, C, D), but not in the population aged < 25 (All-cause mortality: *P* for log-rank test = 0.871, cardiovascular mortality:* P* for log-rank test = 0.472, Additional file 1: Figure S1E, F) and 65–85 (survival analysis curves of the population aged over 65 in Fig. 6C, D).

### Associations of triglyceride glucose index with all-cause and cardiovascular mortality after stratification by age

Based on Figs. 4 and 5, the association of the TyG index with all-cause and cardiovascular mortality was further explored in different age groups. The study population was stratified into two groups based on their age (< 65 vs. ≥ 65). The results showed that TyG index levels were associated with mortality only in the < 65 age group [all-cause mortality: 1.382 (1.214, 1.575), *P*-interaction < 0.001; cardiovascular mortality: 1.772 (1.388, 2.262),* P*-interaction < 0.001]. Furthermore, the study population was grouped into four groups according to age (< 25, 25–44, 45–64, 65–85). TyG index levels were associated with mortality only in the 45–64 age group (all-cause mortality: 1.405 (1.221, 1.617), *P*-interaction = 0.005; cardiovascular mortality: 1.816 (1.402, 2.352), *P*-interaction = 0.002) (Table 3).

**Table 3:** Subgroup analysis for all-cause and cardiovascular mortality according to TyG index after stratification by age

	HR	95% CI	*P*-value	*P* for interaction
All-cause mortality				
Age				
Two groups				< 0.001
< 65	1.382	1.214, 1.575	< 0.001	
≥ 65	1.051	0.947, 1.167	0.350	
Four groups				0.005
< 25	1.718	0.664, 4.583	0.280	
25–44	1.353	0.999, 1.832	0.051	
45–64	1.405	1.221, 1.617	< 0.001	
65–85	1.055	0.950, 1.171	0.320	
Cardiovascular mortality				
Age				
Two groups				< 0.001
< 65	1.772	1.388, 2.262	< 0.001	
≥ 65	0.971	0.790, 1.193	0.777	
Four groups				0.002
< 25	–		–	
25–44	1.518	0.824, 2.799	0.181	
45–64	1.816	1.402, 2.352	< 0.001	
65–85	0.974	0.792, 1.198	0.803	

Adjusted for age, gender, race, BMI, SBP, DBP, TC, uric acid, DM, education, smoking, lipid-lowering agents and hypoglycemic agent

*TyG Index* triglyceride glucose index, *BMI* body mass index, *DBP* diastolic pressure, *SBP* systolic pressure, *TC* cholesterol, *DM* diabetes mellitus, *HR* Hazard Ratio

**Fig. 7:**
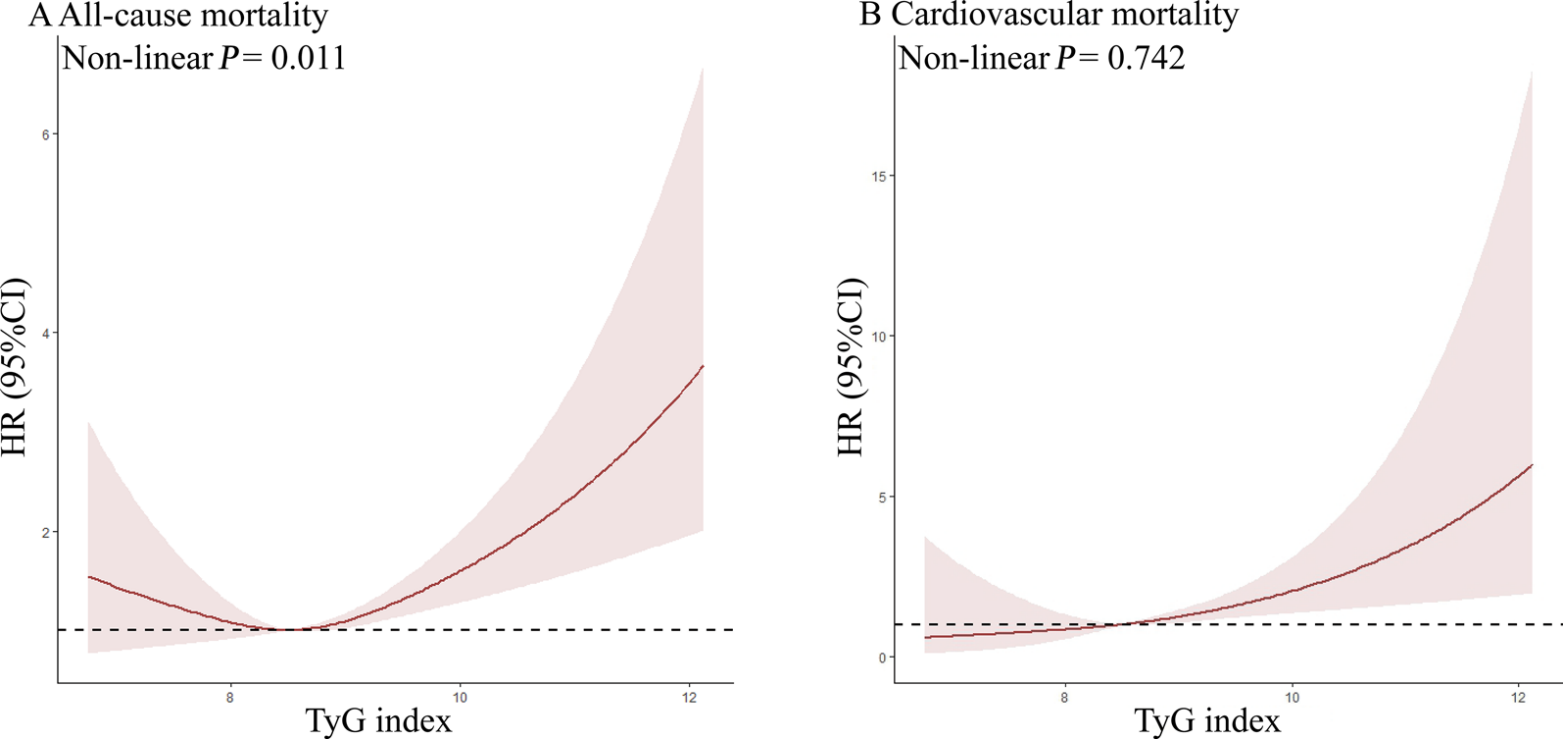
Association between TyG index and all-cause and cardiovascular mortality in aged < 65. **A** all-cause mortality, **B** cardiovascular mortality. Each hazard ratio was computed with a TyG index level of A 8.889 and B 8.781 as the reference. Adjusted for age, gender, race, BMI, SBP, DBP, TC, uric acid, DM, education, smoking, lipid-lowering agents and hypoglycemic agent. *TyG Index* triglyceride glucose index, *BMI* body mass index, *DBP* diastolic pressure, *SBP* systolic pressure, *TC* cholesterol, *DM* diabetes mellitus, *HR* Hazard Ratio

**Fig. 8:**
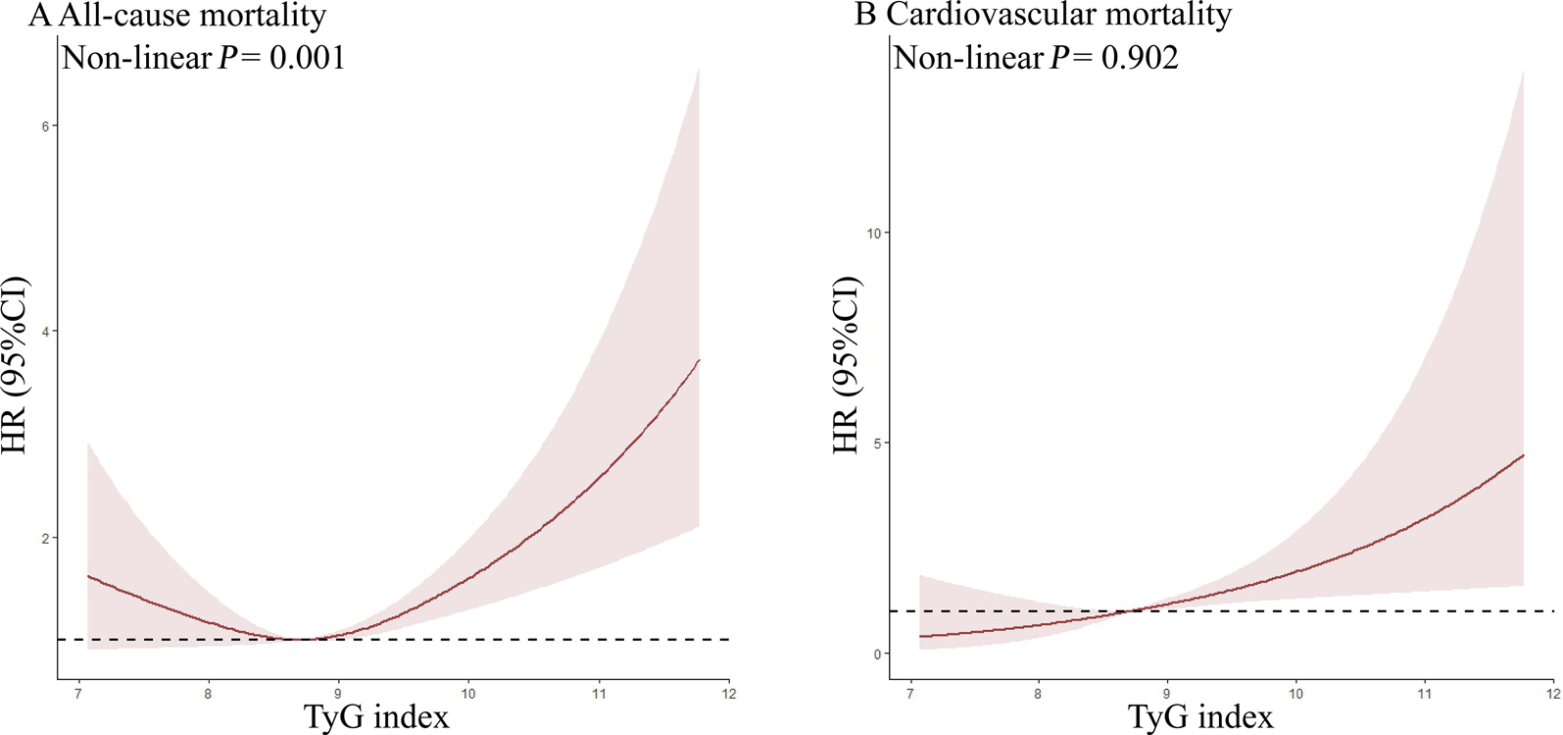
Association between TyG index and all-cause and cardiovascular mortality in aged 45–64. **A** all-cause mortality, **B** cardiovascular mortality. Each hazard ratio was computed with a TyG index level of A 9.008 and B 8.701 as the reference. Adjusted for age, gender, race, BMI, SBP, DBP, TC, uric acid, DM, education, smoking, lipid-lowering agents and hypoglycemic agent. *TyG Index* triglyceride glucose index, *BMI* body mass index, *DBP* diastolic pressure, *SBP* systolic pressure, *TC* cholesterol, *DM* diabetes mellitus, *HR* Hazard Ratio

There was a linear relationship (Non-linear *P* = 0.742, Fig. 7A) and upward trends (*P* for trend = 0.010, Table 4) between the TyG index and cardiovascular mortality in the < 65 age group. Similar results were also observed in the population aged 45–64 (Non-linear *P* = 0.902, Fig. 8A; *P* for trend = 0.015, Table 4). However, the association of the TyG index with all-cause mortality exhibited a non-linear pattern in the < 65 and 45–65 age groups (< 65: Non-linear *P* = 0.011, Fig. 7B; 45–64: Non-linear *P* = 0.001, Fig. 8B).

**Table 4:** Adjusted Hazard ratios (HRs) for all-cause and cardiovascular mortality according to TyG index in aged < 65 and 45–64

	TyG index				*P* for trend
	Quartile 1	Quartile 2	Quartile 3	Quartile 4	
All-cause mortality					
Age					
< 65	1	0.754 (0.529, 1.074)	0.822 (0.582, 1.161)	1.051 (0.746, 1.481)	0.139
45–64	1	0.829 (0.595, 1.156)	0.807 (0.579, 1.123)	1.181 (0.852, 1.637)	0.137
Cardiovascular mortality					
Age					
< 65	1	0.775 (0.306, 1.961)	1.076 (0.453, 2.555)	1.739 (0.750, 4.033)	0.010
45–64	1	1.104 (0.493, 2.473)	1.384 (0.642, 2.984)	2.081 (0.982, 4.409)	0.015

Adjusted for age, gender, race, BMI, SBP, DBP, TC, uric acid, DM, education, smoking, lipid-lowering agents and hypoglycemic agent

Quartile grouping based on TyG index levels for each age group

*TyG Index* triglyceride glucose index, *BMI* body mass index, *DBP* diastolic pressure, *SBP* systolic pressure, *TC* cholesterol, *DM* diabetes mellitus, *HR* Hazard Ratio

### ROC curve analysis of TyG index and HOMA-IR

**Fig. 9:**
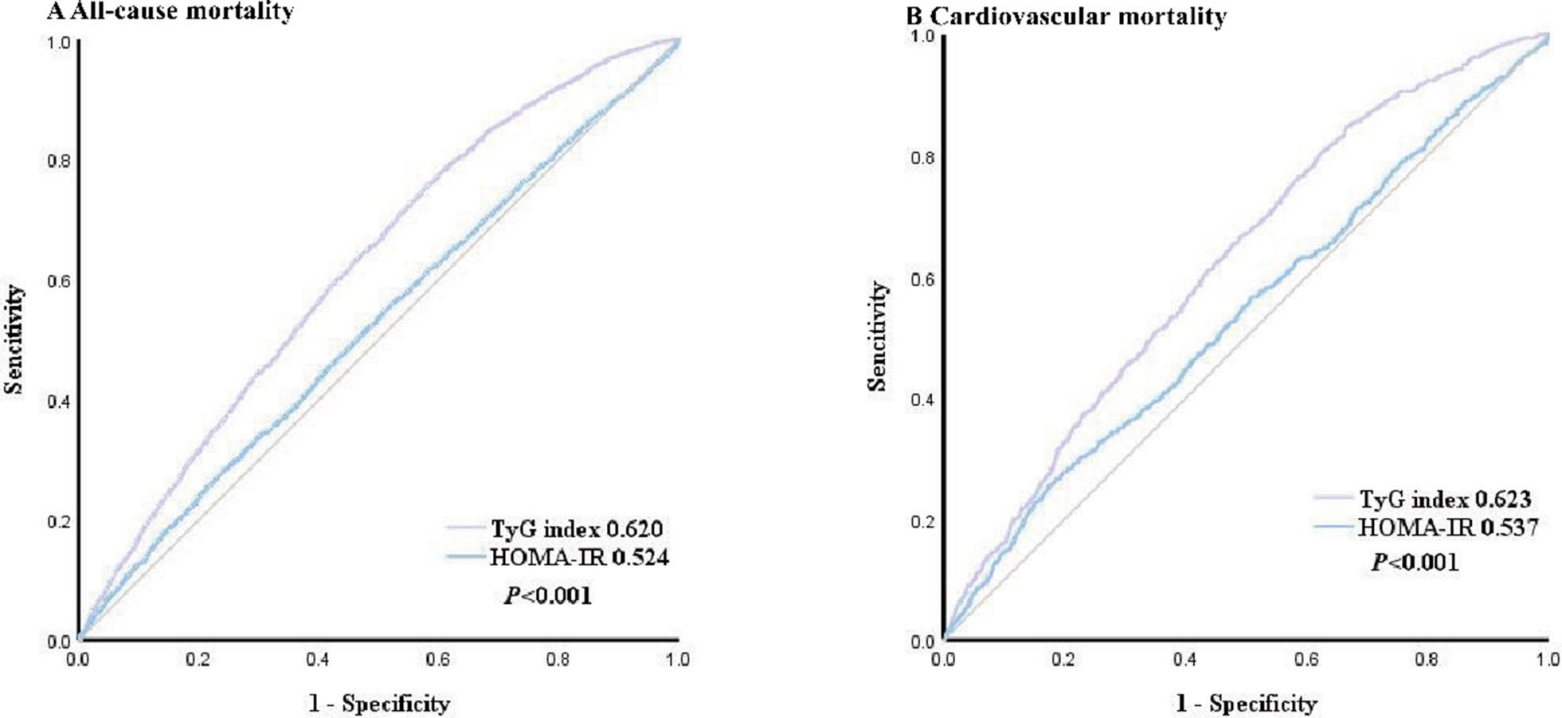
ROC Curve analysis for TyG index and HOMA-IR Predicted All-cause and Heart disease-specific mortality. **A** All-cause mortality; **B** Cardiovascular mortality. *TyG index* triglyceride glucose index, *AUC* area under curve, *ROC* receiver operating characteristic, *HOMA-IR* homeostasis model assessment of insulin resistance

The ROC curve for the TyG index and HOMA-IR in predicting primary endpoints is depicted in Fig. 9. As expected, the TyG index outperformed HOMA-IR in predicting all-cause mortality (0.620 vs. 0.524, *P* < 0.001). Similar results were also observed in predicting cardiovascular mortality (0.623 vs. 0.537,* P* < 0.001).

The cut-off values for the TyG index to predict all-cause and cardiovascular mortality were 8.401 and 8.289, respectively. The sensitivity was 0.757 and 0.848, while the specificity was 0.420 and 0.335, respectively.

## Discussion

This study revealed a significant association between the TyG index and all-cause as well as cardiovascular mortality in the general population, demonstrating its superiority over HOMA-IR for predicting these outcomes. Notably, a substantial interaction effect was observed between the TyG index and age, indicating that the relationship between TyG levels and mortality was most prominent in younger individuals. Specifically, this association was particularly robust in the 45–64 age group but relatively weaker in older populations. Moreover, while a linear relationship was established between the TyG index and cardiovascular mortality in those aged < 65, a non-linear pattern was observed in the case of all-cause mortality.

Our findings of a non-linear association between TyG index and all-cause and cardiovascular mortality in the overall population were consistent to some extent with recent studies conducted by Yu et al. and Liu et al. [12, 17]. Liu and his colleagues observed a non-linear association between the TyG index and mortality, both for all-cause and cardiovascular disease [17]. In another study, Yu and his colleagues reported a U-shaped relationship between the TyG index and all-cause and cardiovascular mortality [12]. Notably, they did not perform age stratification, whereas our study stratified patients according to age, revealing that the TyG index exhibited a significant association with mortality only in individuals aged under 65, particularly in the 45–64 age group. Furthermore, our study demonstrated that the TyG index maintained its non-linear association with all-cause mortality while displaying a linear association with cardiovascular mortality in those aged under 65, especially in the 45–64 age bracket. On the other hand, Kim and his colleagues observed that a higher TyG index was linked to an increased risk of all-cause mortality (HR 1.12; 95% CI 1.03–1.22) and cardiovascular disease (HR 1.26; 95% CI 1.02–1.55) in patients with normal glucose tolerance [11]. In our study, we observed an upward trend in the relationship between the TyG index and cardiovascular mortality in the 45–65 age group, while the positive association with all-cause mortality was not as pronounced. Liu and his colleagues conducted a meta-analysis and reported no significant association between the TyG index and mortality [10]. Nevertheless, due to heterogeneity in study population, follow-up duration, and sample size, the precise relationship between the TyG index and all-cause and cardiovascular mortality in the general population remains uncertain.

In the present study, we also observed an age-related difference in the association between the TyG index and mortality, consistent with previous research in patients with cardiovascular disease and ischemic stroke. Ma and his colleagues discovered that participants with higher TyG index levels had a greater incidence of major cardiovascular adverse events in middle-aged patients, but not in older patients [14]. Likewise, cardiovascular disease patients under the age of 65 exhibited higher TyG levels and a greater risk of all-cause mortality [16]. Similar trends were identified in stroke patients, with the higher TyG index group having a significantly increased risk of mortality among younger patients, while the association was less prominent among patients over 65 years old. Additionally, an interaction was observed between the TyG index and age in relation to mortality [15]. In contrast, Liu and his colleagues found that the TyG index was associated only with all-cause mortality among participants aged 65 and older. This discrepancy might be attributed to varying research objectives, given that we focused on examining the relationship between the TyG index and mortality in different age subgroups, while Liu and his colleagues may have been primarily concerned with identifying age subgroups where specific thresholds applied [17].

The results of our study indicated a non-linear correlation between the TyG index and all-cause mortality, revealing a positive connection between the TyG index and cardiovascular mortality in individuals under the age of 65, specifically within the age range of 45–64. Although previous studies have demonstrated that higher TyG index levels were associated with a higher risk of all-cause mortality under the age of 65. It is crucial to acknowledge that these investigations predominantly focused on patients suffering from cardiovascular or cerebrovascular conditions [15, 16], suggesting that the observed all-cause mortality outcomes were inherently influenced by these diseases. As a result, the specific proportion of cardiovascular mortality within these studies remained unclear. Hence, the aforementioned findings did not provide a comprehensive explanation for the features of correlation between the TyG index and overall mortality in the general population aged under 65. And our research contributes to addressing this point. In addition, our research finding was partially consistent with the recent research conducted by Professor Patricio Lopez Jaramillo and colleagues, who conducted a comprehensive prospective cohort study of participants aged 35–70 [20]. Their study revealed that individuals in the highest tertile of the TyG index exhibited elevated risks for cardiovascular mortality. In addition, they also found that no significant association of the TyG index was seen with non-cardiovascular mortality. Our study did not encompass non-cardiovascular mortality, and it remains uncertain whether the non-linear correlation between TyG and all-cause mortality was influenced by non-cardiovascular mortality. Additional investigation is warranted.

Little is currently known about the mechanism underlying the correlation between the TyG index and mortality. This association, in our view, can be attributed to the TyG index's ability to reflect the level of IR in patients. Insulin resistance is known to trigger the formation of glycosylation products and free radicals, which contribute to a reduction in the bioavailability of nitric oxide (NO). Consequently, this process damages the vascular endothelium, impairs endothelium-dependent vasodilation, and precipitates the onset of various diseases [21]. Moreover, IR triggers the mitochondrial electron transport chain, resulting in excessive reactive oxidative stress and subsequent damage to the endothelium. Furthermore, IR can cause a metabolic imbalance in individuals with diabetes [22], leading to hyperglycemia, inflammation, oxidative stress, and disruptions in lipid metabolism [23, 24]. Notably, insulin resistance itself serves as a crucial marker for obesity, hypertension, dyslipidemia, and other metabolic syndromes, which are intricately linked to cardiovascular diseases and mortality [5].

The significant association between TyG levels and all-cause and cardiovascular mortality in the general population was predominantly observed in non-older groups, particularly in the 45–64 age bracket, rather than in the older population. The exact reasons for such a pattern remain subject to ongoing debate. It is widely thought that younger individuals are more prone to developing insulin resistance [15, 25], resulting in elevated TyG levels in this demographic [26–28]. Additionally, it is highly conceivable that the prolonged duration of the disease in young patients may result in increased exposure to insulin resistance, potentially leading to more aggressive complications. While there is growing research on the incidence rates of early-onset type 2 diabetes and its complications [29–31], there is still a dearth of studies examining whether the incidence rates of insulin resistance and its complications in young individuals are consistent and whether the incidence rates of insulin resistance complications in the younger population surpass those in older cohorts. Notably, the measurement of the TyG index in older individuals is susceptible to more confounding factors, such as the presence of various illnesses, accompanying poor nutritional status, and altered blood lipid levels. Consequently, the TyG index may not serve as an accurate reflection of insulin resistance in older individuals compared to their younger counterparts [32].

## Strengths and limitations

This study represents a significant advancement in the field by proposing the potential modifying effect of age on the TyG index in predicting all-cause and cardiovascular mortality in the general population, thereby building upon previous research. Moreover, our study highlighted a robust association between the TyG index and all-cause as well as cardiovascular mortality in individuals aged under 65, particularly within the 45–64 age group. This information provides the theoretical foothold for the application of the TyG index in the non-older population. Notably, the study benefited from utilizing a large-scale national database with a substantial sample size and a prolonged follow-up period. Despite these strengths, several limitations present in our study should be acknowledged. Firstly, the post hoc nature of the analysis meant that residual confounding factors could not be entirely mitigated. Secondly, the study did not involve dynamic monitoring of changes in the TyG index. Thirdly, the results were derived from the general population in the United States, necessitating caution when extrapolating findings to other racial and demographic groups. In conclusion, future research should consider these limitations in their investigations.

## Conclusion

The TyG index exhibited a significant association with all-cause and cardiovascular mortality in the general population, especially among individuals aged under 65. Notably, a non-linear relationship was observed between the TyG index and all-cause mortality in the under-65 age group, while a positive correlation was noted with cardiovascular mortality.

### Supplementary Information

**Additional file 1: Figure S1.** Kaplan–Meier survival analysis curves for all-cause and cardiovascular mortality after stratification by age. A Kaplan–Meier analysis for all-cause mortality among TyG index groups in (A) < 25 years old, (C) 25–44 years old, (E) 45–64 years old. A Kaplan–Meier analysis for cardiovascular mortality among TyG index groups in (B) < 25 years old, (D) 25–44 years old, (F) 45–64 years old.

## Data Availability

The data that support the findings of this study are available from the corresponding author.
