# Supplementary material for: Association of triglyceride glucose index with all-cause and cardiovascular mortality in the general population
Source: Cardiovasc Diabetol. 2023 Nov 22;22:320. doi: 10.1186/s12933-023-02054-5 (PMC10666367; doi:10.1186/s12933-023-02054-5)
Supplement: Supplementary file 1 — Additional file 1: Figure S1. Kaplan–Meier survival analysis curves for all-cause and cardiovascular mortality after stratification by age. A Kaplan–Meier analysis for all-cause mortality among TyG index groups in (A) < 25 years old, (C) 25–44 years old, (E) 45–64 years old. A Kaplan–Meier analysis for cardiovascular mortality among TyG index groups in (B) < 25 years old, (D) 25–44 years old, (F) 45–64 years old. [file 12933_2023_2054_MOESM1_ESM.docx]

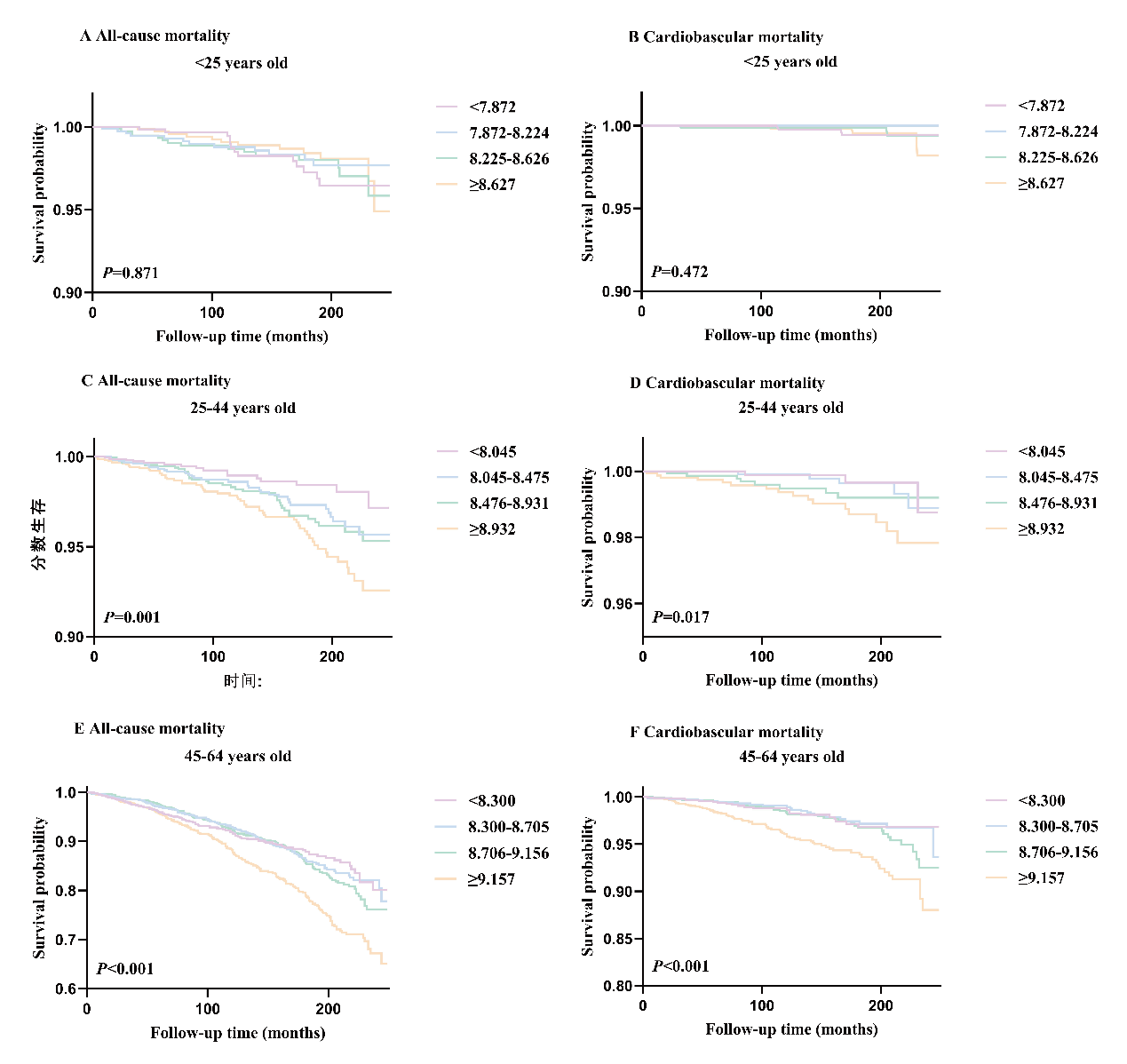


Additional file Figure 1 Kaplan–Meier survival analysis curves for all-cause and cardiovascular mortality after stratification by age

A Kaplan–Meier analysis for all-cause mortality among TyG index groups in (A) <25years old, (C) 25-44 years old, (E) 45-64 years old. A Kaplan–Meier analysis for cardiovascular mortality among TyG index groups in (B) <25years old, (D) 25-44 years old, (F) 45-64 years old.
